# Impact of Alzheimer’s Disease on Caregiver Questionnaire: internal consistency, convergent validity, and test-retest reliability of a new measure for assessing caregiver burden

**DOI:** 10.1186/s12955-014-0114-3

**Published:** 2014-09-04

**Authors:** Jason C Cole, Diane Ito, Yaozhu J Chen, Rebecca Cheng, Jennifer Bolognese, Josephine Li-McLeod

**Affiliations:** Covance Market Access Services Inc, 10300 Campus Point Drive, Suite 225, San Diego, CA 92121 USA; Baxter Healthcare Corporation, One Baxter Way, Westlake Village, CA 91362 USA; Covance Market Access Services Inc, 9801 Washingtonian Blvd., 9th Floor, Gaithersburg, MD 20878 USA; Current address: Pharmaceutical Product Development, 9330 Scranton Road, Suite 200, San Diego, CA 92121 USA

**Keywords:** Alzheimer’s disease, Caregivers, Burden, Psychometrics, Questionnaire

## Abstract

**Background:**

There is a lack of validated instruments to measure the level of burden of Alzheimer’s disease (AD) on caregivers. The Impact of Alzheimer’s Disease on Caregiver Questionnaire (IADCQ) is a 12-item instrument with a seven-day recall period that measures AD caregiver’s burden across emotional, physical, social, financial, sleep, and time aspects. Primary objectives of this study were to evaluate psychometric properties of IADCQ administered on the Web and to determine most appropriate scoring algorithm.

**Methods:**

A national sample of 200 unpaid AD caregivers participated in this study by completing the Web-based version of IADCQ and Short Form-12 Health Survey Version 2 (SF-12v2™). The SF-12v2 was used to measure convergent validity of IADCQ scores and to provide an understanding of the overall health-related quality of life of sampled AD caregivers.

The IADCQ survey was also completed four weeks later by a randomly selected subgroup of 50 participants to assess test-retest reliability. Confirmatory factor analysis (CFA) was implemented to test the dimensionality of the IADCQ items. Classical item-level and scale-level psychometric analyses were conducted to estimate psychometric characteristics of the instrument. Test-retest reliability was performed to evaluate the instrument’s stability and consistency over time.

**Results:**

Virtually none (2%) of the respondents had either floor or ceiling effects, indicating the IADCQ covers an ideal range of burden. A single-factor model obtained appropriate goodness of fit and provided evidence that a simple sum score of the 12 items of IADCQ can be used to measure AD caregiver’s burden. Scales-level reliability was supported with a coefficient alpha of 0.93 and an intra-class correlation coefficient (for test-retest reliability) of 0.68 (95% CI: 0.50–0.80). Low-moderate negative correlations were observed between the IADCQ and scales of the SF-12v2.

**Conclusions:**

The study findings suggest the IADCQ has appropriate psychometric characteristics as a unidimensional, Web-based measure of AD caregiver burden and is supported by strong model fit statistics from CFA, high degree of item-level reliability, good internal consistency, moderate test-retest reliability, and moderate convergent validity. Additional validation of the IADCQ is warranted to ensure invariance between the paper-based and Web-based administration and to determine an appropriate responder definition.

## Background

Alzheimer’s disease (AD) is an age-related, irreversible, progressive brain disorder that attacks the brain and results in increasingly impaired memory, thinking, reasoning, and behavior [1,2]. The prevalence of AD is estimated at 5.4 million in the United States (US) and as high as 24 million globally [3,4]. There were an estimated 454,000 new cases diagnosed in 2010 [5]. Barring significant medical breakthroughs, prevalence rates are predicted to triple by 2050 [6].

The cognitive impairments from AD significantly impact the patient’s activities of daily living [7]. Caregiving is an inherent part of managing AD, as progressive deterioration in intellectual function and other cognitive skills leads to a decline in the ability to perform activities of daily living (ADLs) [7]. Caring for patients with AD poses a large burden on both families and the healthcare community [5]. Over 35 million people worldwide currently live with Alzheimer’s disease, and this number is expected to double by 2030 and more than triple by 2050 to 115 million. In the 2010 World Alzheimer Report, Alzheimer’s Disease International estimated that the annual societal costs of dementia worldwide were US $604 billion, or 1% of the aggregated worldwide gross domestic product. Alzheimer’s Disease International also predicted almost a doubling in worldwide societal costs from US $604 billion in 2010 to US $1,117 billion by 2030 [6]. In the 2010 World Alzheimer Report, a systematic review of the world literature on the demands of caregiving looked at 10 studies where time spent assisting with basic ADLs was quantified covering 25 countries; 13 studies of time spent in generally supervising the person with dementia covering 25 countries; and 42 studies of time spent assisting with basic ADLs and instrumental activities of daily living (IADLs) combined spanning 30 countries. The report suggested that caregivers spend an average of 2.0 hours daily supporting basic ADLs, 3.6 hours with basic ADLs and IADLs combined, and 2.6 hours supervising the person with dementia. This amounts to an average weekly total of between 14 hours (ADL alone) and 43 hours (ADL, IADL, and supervision) [6]. In 2011, 15.2 million family and other uncompensated caregivers cared for patients with AD and other dementias in the US providing 17.4 billion hours of care valued at more than $210 billion [5]. Family caregivers provide 80% of home care to AD patients with the level of caregiver burden related to the extent of the patient’s cognitive impairment and functional abilities [5,6]. Caregivers provided an average of 21.9 hours of care per week [6].

Given the high demands on caregivers of people with AD, they may experience negative impacts on physical, psychological, emotional, social, and financial aspects of their life; some positive effects have also been noted [8-11]. About a third of family caregivers experience symptoms of depression, and 61% rate the emotional stress of caregiving as high or very high [4]. They may rate their own health as fair or poor and tend to report that serving as a caregiver worsens their health [4]. Caregivers may also experience higher levels of depression and stress hormones, reduced immune function, slow wound healing, and more new cases of hypertension and coronary heart disease compared to non-caregivers [12]. Health care costs for caregivers are estimated to be 8% higher than non-caregivers due to the physical and emotional toll of caregiving [4]. Caregiver healthcare is estimated to cost $8.6 billion; this is in addition to the $210 billion in unpaid caregiver hours [5]. Caregiving also negatively impacts employment status and work productivity. Of the 44% of caregivers who are employed part or full time, 65% reported missing work, going in late, or leaving early, and 20% have taken a leave of absence [5]. The burden of caregiving and resulting changes in employment often lead to withdrawal or isolation from the caregiver’s wider social networks, which may further increase depression and stress [12-14].

Although private or public insurance offers partial coverage, providing care often results in out-of-pocket expenditures for the family. In 2008, total per-person payments from all sources for health care and long-term care for Medicare beneficiaries with AD and other dementias were 3 times as great as payments for other Medicare beneficiaries in the same age group [5]. Excluding the contributions of uncompensated caregivers, total payments for care related to AD and dementia care for patients 65 years and older were estimated at $200 billion in 2012 [5].

A caregiver burden instrument specific to AD is necessary to measure the impact of treatment for AD patients on the lives of their caregivers. The ideal instrument would be brief, self-administered, and not overly burdensome to the respondent. A review of the existing caregiver burden instruments in the published literature revealed a range of validated and non-validated instruments measuring various aspects of the caregiver experience, including burden, mood, needs, and quality of life, across a range of conditions. In a systematic review of caregiver burden instruments, Deeken et al. [15] identified 28 self-report questionnaires assessing the burden, needs, and quality of life of informal caregivers. Among the 17 instruments reviewed that specifically focused on caregiver burden, 10 of the instruments were not specific to either AD or dementia (eg, Caregiver Strain Index [16], Burden Assessment Scale [17], Appraisal of Caregiving Scale [18]); several lacked evidence of rigorous psychometric testing or evidence of adequate validity and reliability (eg, Burden Interview [19], Family Burden Scale [20], Objective Burden Questionnaire [21]; and others were too lengthy or difficult to administer in a clinical trial setting (eg, Caregiver Experience Assessment, 105 items [22]; Caregiver Stress Inventory, 43 items [23]). As a result, despite the abundance of caregiver instruments available, none were appropriate for the purposes of measuring caregiver burden during the course of a clinical trial evaluating the impact of an experimental treatment on AD patients. In addition, the Zarit Burden Interview was assessed but found to be too lengthy to be used in an Alzheimer’s Disease interventional trial where the caregiver was asked to complete a number of psychosocial measures on behalf of the AD patient [10].

### Development of Impact of Alzheimer’s Disease on Caregiver Questionnaire (IADCQ)

In response to the lack of an appropriate AD caregiver instrument for clinical trials, an effort was undertaken to develop a new AD caregiver instrument. A review of the literature on the health-related quality of life (HRQoL) burden that unpaid caregivers face in caring for an individual with AD was first conducted. A Medline search was conducted using Alzheimer, dementia, caregiver burden, and caregiver quality of life as search terms from 1980 to 2010. Nineteen articles were found to be relevant. Eight articles discussed AD caregiver burden in general and 11 existing instruments were identified. However, none of these findings fulfilled the criteria of assessing AD caregiver burden for various reasons such as inability to implement the instrument in a clinical trial setting, inappropriate questions, too lengthy of an instrument, and not a self-administered instrument.

Based on this review, the initial draft of the Impact of Alzheimer’s Disease on Caregiver Questionnaire (IADCQ), consisting of 9 items, was created. To ensure that this questionnaire adequately captured the key domains that were most relevant to caregivers of AD patients in assessing the burden of caregiving, 3 focus groups of 21 unpaid caregivers (21 females and 2 males) of AD patients were held in Los Angeles, Chicago, and New Orleans. These focus groups were held to better understand the experience of caring for a patient with AD and to conduct a cognitive debriefing of the initial draft of the IADCQ. The focus groups were held to elicit concepts and were conducted with a trained moderator using a semi-structured interview guide. Caregivers described various impacts on their HRQoL due to caregiving which included emotional (worry, frustrated, sad/depressed), social (relationship with friends and family, relationship with person with AD, limit activities), physical (aging, diet, weight), sleep (falling asleep, less sleep, interruption), work (can’t retire, work at home, cut back on hours worked), time (having no time to do personal activities, giving up time to care, making adjustments to schedule), sex life, well-being (lack of freedom/independence, loss of creativity, needing to mature faster, loss of self, personality change), and financial. Caregivers also reported not knowing what to expect and needing to make decisions for the patient. They reported that their self-care was impacted and they felt homebound. Caregivers were asked to evaluate the initial draft of the IADCQ and provide input on the questions, response options, and instructions. Saturation was achieved by the third focus group. Based on the results, a revised 12-item IADCQ instrument with a 7-day recall period was developed. This instrument contained the elements most relevant to caregivers of AD patients in assessing the burden of care giving: emotional, physical, social, time, sleep, and financial impact.

The current study details the next steps in IADCQ development, including a psychometric study of the IADCQ and ascertaining the most appropriate scoring algorithm for the instrument.

## Methods

### Study design

The current study design and psychometric analyses were selected to establish the internal consistency and test-retest reliability of the IADCQ. Alzheimer’s disease caregivers were recruited and entered into a cross-sectional study to collect data appropriate for most of the study goals. Finally, to ascertain test-retest reliability, a subset of those who completed the psychometric study was randomly invited to participate in a second round of data collection four weeks later.

Psychometric analyses were specifically selected and ordered to ensure goals of the study were analyzed accurately. The IADCQ was first analyzed with confirmatory factor analysis (CFA) to assure construct validity; the data were checked for fit with the original conceptual framework from previous qualitative development of the IADCQ. The reliability (eg, internal consistency) was examined by assessing item-level and scale-level statistics. Finally, test-retest reliability was examined with intra-class correlations to determine the strength of the relationship between four-week administrations of the IADCQ. Pearson correlation coefficients were computed for the investigation of convergent validity.

### Study participants

A national sample of men and women ≥ 18 years of age who identified themselves as an unpaid caregiver of an AD patient participated in a cross-sectional, nonrandomized, psychometric study. The AD caregivers, who previously indicated their willingness to be contacted for research purposes, were recruited via e-mail from a panel of caregivers in the US managed by a research-panel vendor. Each caregiver previously self-enrolled to participate in research related to caregiving for AD patients. Various approaches were employed to recruit panelists, such as banners, referrals, natural search optimization, affiliate marketing, and targeted e-mails. Inclusion and exclusion criteria (as completed by self-report), outlined in Table 1, were employed to determine the survey candidates’ eligibility. If caregivers were interested and eligible to participate in the study, they read and provided informed consent electronically before completing the demographic questions and the study instrument. Each initial e-mail was submitted with a unique link. When the caregiver noted they were willing to participate, the system created a follow-up email at the correct time with the same caregiver ID in the secure link. The caregivers were compensated for participating in the study. This study was reviewed and approved by the New England Institutional Review Board.

**Table 1:** **Inclusion and exclusion criteria to recruit for survey participants**

**Inclusion criteria**		**Exclusion criteria**	
**Age**	≥ 18 years	**Questionable validity of content**	Each participant was required to read the informed consent form that appeared on their computer screen prior to beginning the survey.
**Alzheimer’s disease caregiver status**	Informal caregiver to an AD patient	**Cognitively impaired**	Possess any of the following conditions:
			psychiatric disorder,
			developmental disorder that affected cognitive or emotional functions so that judgment and reasoning were significantly diminished,
			under the influence of/dependent on drugs/alcohol, or
			suffering from degenerative diseases affecting the brain.
**English literacy**	Able to read and write English sufficiently to complete the study instrument and provide the informed consent.		

A total of 200 caregivers completed the online survey. To assess the test-retest reliability of the IADCQ, a subgroup of 50 randomly selected caregivers were asked to repeat the survey four weeks after the initial survey; 100% of these caregivers completed the second administration.

### Measurements

#### Description of the IADCQ

The Impact of Alzheimer’s Disease on Caregiver Questionnaire (IADCQ) is an instrument used to measure the burden of caregiving and includes items that represent the key concepts and domains of caregiving for an AD patient. The current version of the IADCQ has a 7-day recall period and 12 items. It has a five-point Likert scale with response choices ranging from “not at all” (0) to “extremely” (4). The IADCQ measures the burdens associated with being an AD caregiver across six theorized domains: emotional, physical, social, financial, sleep, and impact on time.

The original IADCQ qualitative research [24] helped to gain a better understanding of the impact of AD patient caring on caregivers’ HRQoL. The current study is the first quantitative evaluation of the IADCQ and was designed firstly to evaluate the psychometric characteristics of a Web-based version of the IADCQ instrument completed by caregivers of patients with AD and secondly, to determine the scoring algorithm.

A comprehensive examination of the psychometric properties of the IADCQ was undertaken in a group of caregivers for AD patients through a Web-based survey. The survey was administered twice: (1) at baseline (using the IADCQ and Short Form-12 Health Survey Version 2 [SF-12v2™]) and (2) four weeks later for a subgroup of participants (using the IADCQ).

#### Description of the SF-12v2

In addition to the IADCQ, the SF-12v2 was administered in the survey. The SF-12v2 is a generic HRQoL instrument that contains 12 questions representing 8 domains to provide insight into physical and mental functioning [25]. It is a valid measure of physical and mental health often used in large population health surveys or in clinical trials to assess the impact of an intervention on patient HRQoL. It was used to permit a wide array of HRQoL information, and its psychometric properties are well defined and known. In addition, it has accepted responder definitions. In this study, the SF-12v2 was used to measure convergent validity of IADCQ scores as well as to provide an understanding of the overall HRQoL of the sampled AD caregivers.

### Data analysis

Descriptive statistics were first examined based on demographics (ie, age, gender, and race) and other characteristics of the AD caregiver participants (ie, caregiver history, employment status, and missing work time). Item-level evaluations were assessed to cover aspects such as completeness of responses, response choices used by participants, distribution of responses, and ordering of item means. The Web-based survey did not permit missing data; therefore, all 200 subjects had complete survey data. Survey items were defined to assess a specified level within a narrow range of the construct for instrument precision, with some participants scoring the lowest possible score (ie, floor effects) and others having the highest possible score (ie, ceiling effects).

The sequence of psychometric analyses in this study was designed to ensure proper understanding of the latent structure before performing the classical psychometric analyses. The IADCQ latent structure was evaluated with CFA to assure that the IADCQ scoring matched with the conceptual framework. We then evaluated the psychometric characteristics of the measures using classical psychometric techniques and examined the instrument properties by assessing item-level and scale-level statistics. All analyses, unless otherwise specified, were conducted using Statistical Analysis Software (SAS) version 9.1.

#### Latent structure analyses

Confirmatory factor analysis was used to investigate the dimensionality of the IADCQ instrument and to ensure that the scoring approach matches with the latent structure of the IADCQ. Through an earlier unpublished qualitative study, six domains in the IADCQ were identified. Latent analyses were designed to determine whether the hypothesized organization of items to domains was consistent with the empirically tested latent structure of the IADCQ. The rationale for this analysis was to measure the extent to which the scoring system explains the way that caregivers respond to the items in the IADCQ to provide evidence for the structural fidelity of the scoring system fitting with the latent constructs underlying the IADCQ.

The aggregate data of item responses from the IADCQ were submitted to CFA appropriate for categorical data. Specifically, we used a parametric extraction of maximum likelihood but subjected the covariance matrix to bootstraps to correct for the influence of non-normality [26]. Two thousand Bollen-Stine bootstraps were used during the model estimation to control for multivariate non-normality. Model fit statistics in CFA provided the measures with the strength of relationship between the theoretical model and the data: (1) goodness of fit index (GFI; measures the amount of variance and covariance in the data that are reproduced by the tested model); (2) comparative fit index (CFI; specifies the amount of difference between the examined model and the independence model); (3) non-normed fit index (NNFI; conducts the same task as CFI but takes into consideration the number of parameters in a model—an aspect that can inflate CFI); (4) root mean square error of approximation (RMSEA; determines how well the examined model reproduces the saturated model); and (5) standardized root mean square residual (SRMR; similar to RMSEA but specifies the absolute measure of model fit). The model would be considered satisfactory if the five fit indices met or surpassed these thresholds: GFI ≥ 0.90 [27], CFI and NNFI ≥ 0.95 [27], RMSEA ≤ 0.06 [28], and SRMR ≤ 0.08 [29]. Confirmatory factor analysis was conducted using Mplus Version 6.1, a latent variable modeling program [30].

#### Item-level analyses

For the item-level psychometric evaluation, five sets of analyses were planned after the latent analysis following the methods described in Cole et al. [31]: (1) equality of item-total correlations for each scale (highest vs. lowest item-total correlation *p* > 0.05); (2) equality of variances for each item per scale (Hartley’s F_max_ < 3.0); (3) sufficient item-total correlations (≥0.40); (4) small alpha removed statistics (≤0.02); and (5) item-total correlations that were higher for each item’s own scale than for other scales (*p* < 0.05).

#### Scale-level evaluation

After estimating the item-level psychometrics, the scale-level properties of the IADCQ domains were examined in five aspects: (1) scale means and standard deviations; (2) floor and ceiling scores; (3) internal consistency reliability; (4) test-retest reliability; and (5) convergent validity. Along with providing descriptive statistics (ie, mean and standard deviation) for the IADCQ and SF-12v2 scores, we also assessed the overall floor and ceiling effects of the IADCQ for the purpose of assessing precision of the instrument, and the percentages of participants with the floor or ceiling scores were calculated. Floor and ceiling effects were classified when either was achieved by more than 5% of the sample.

Internal consistency reliability was measured with two techniques: coefficient alpha and average inter-item correlation. For coefficient alpha [32], reliability coefficients of ≥ 90 have been suggested for individual-level analyses [29], though an internal consistency of ≥ 80 is considered to be sufficient for most cases [32]. Internal consistency was also measured with the average inter-item correlation, which should range from between 0.3 (for a general scale) to 0.5 (for a specific scale) [33,34].

Test-retest reliability was conducted through a one-way random effects intra-class correlation coefficient (ICC) to evaluate the reliability and stability of the IADCQ and to assess the consistency of the instrument over time. Because of the short time frame in which the instruments were administered, it was expected that the measures of these constructs would either not change or change minimally. Finally, validity of the IADCQ scale was analyzed via Pearson correlations with baseline scores on the SF-12v2 measuring physical and mental health composite scores (PCS and MCS) scales and subscales for various measures of HRQoL (all correlations were expected to be negative given the inverse relationship of healthy scores on the IADCQ and SF-12v2). The correlations between the IADCQ and the SF-12v2 scores can provide an appropriate measure of overall convergent validity.

## Results

### Participants

A total of 200 AD caregivers (80 males, 120 females) completed the Web-based survey. Overall, 87% of participants were between the ages of 30 and 69 years; 40% were between 30 and 49 years; and 47% were between 50 and 69 years (Table 2). The majority of participants were white, and a third had been caregivers for < 1 year. There were 42.5% of participants employed full time (ie, ≥ 30 hours per week), followed by participants who were employed part time (ie, < 30 hours per week) because of caregiving responsibilities (13%) or retired (13%). Among the caregivers who were employed, the majority of participants had missed zero to five days from work per month due to caregiving duties. Details of the demographic statistics for the test-retest sample are also provided in Table 2.

**Table 2:** **Demographics of the Web-based survey participants**

**Type of characteristics**	**Baseline web-based survey (N = 200)**		**Re-test web-based survey (N = 50)**	
*Age*	n	%	n	%
18–29 years	22	11.0%	6	12.0%
30–49 years	79	39.5%	22	44.0%
50–69 years	94	47.0%	21	42.0%
≥ 70 years	5	2.5%	1	2.0%
*Gender*				
Male	80	40.0%	19	38.0%
Female	120	60.0%	31	62.0%
*Race*				
White	169	84.5%	42	84.0%
Black/African American	14	7.0%	4	8.0%
Asian	9	4.5%	4	8.0%
American Indian/Alaskan Native	2	1.0%	0	0.0%
Other	6	3.0%	0	0.0%
*History being an AD caregiver*				
< 6 months	20	10.0%	2	4.0%
6–12 months	45	22.5%	6	12.0%
13–24 months	51	25.5%	14	28.0%
> 2 years	84	42.0%	28	56.0%
*Employment status*				
Full-time homemaker	16	8.0%	4	8.0%
Employed full time (≥30 hours per week)	85	42.5%	26	52.0%
Employed part time because of caregiving responsibilities (<30 hours per week)	26	13.0%	9	18.0%
Employed part time not because of caregiving responsibilities (<30 hours per week)	11	5.5%	5	10.0%
Unemployed because of caregiving responsibilities	17	8.5%	4	8.0%
Unemployed not because of caregiving responsibilities	11	5.5%	0	0.0%
Volunteer/Student	4	2.0%	0	0.0%
Retired	26	13.0%	1	2.0%
Other	4	2.0%	1	2.0%
*Days per month missed from work due to caregiving duties*				
0–5 days	82	67.2%	22	44.0%
6–10 days	28	22.9%	16	32.0%
11–15 days	8	6.6%	1	2.0%
16–20 days	1	0.8%	1	2.0%
21–24 days	3	2.5%	0	0.0%

Abbreviation: AD, Alzheimer’s disease.

### Confirmatory factor analysis

The initially theorized, six-factor model was not supported by CFA. One of the prefaces in latent modeling is that a single factor should be considered as either the only factor or as an underlying single factor, subsuming all other factors; therefore, it is plausible to examine for their goodness of fit under a default one-factor model [35]. The goodness of fit of the single-factor model to the survey data was evaluated using the GFI, CFI [29], NNFI [36], RMSEA [37], and SRMR [26]. The CFA model obtained fit that reached most of the acceptance thresholds, where GFI = 0.934, CFI = 0.944, NNFI = 0.934, and RMSEA = 0.076 (90% confidence interval [CI]: 0.059–0.090). Although the RMSEA (0.076) was higher than the ideal value of 0.06, a strong SRMR finding (with the value of 0.040) suggested that the amount of free parameters challenged obtaining a favorable RMSEA. The analysis results indicated that a single-factor model obtained appropriate goodness of fit and provided evidence that a simple sum score of the 12 items of the IADCQ can be used to measure AD caregiver burden.

**Figure 1:**
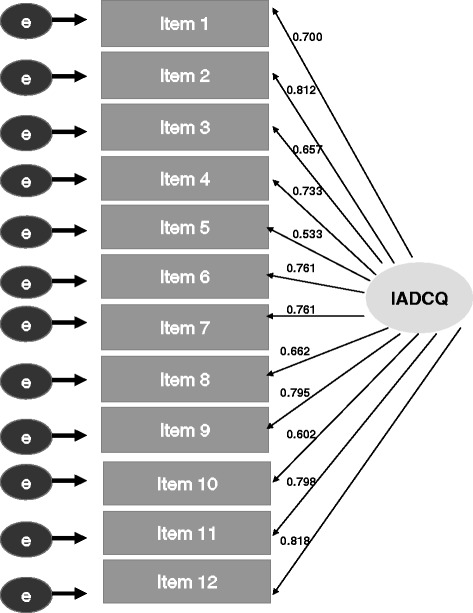
**Finalized CFA model structure and path coefficients for the 12-item IADCQ. **CFA=confirmatory factor analysis; IADCQ=Impact of Alzheimer’s Disease Caregiver Questionnaire; e=residual variance.

The finalized CFA model depicts the strength of the relationship between each latent trait and its reflective items, including the standardized path coefficients for the variables on each of the items, as well as the level of correlation on the factor (Figure 1). High standardized factor loadings were observed for all items on the single factor.

### IADCQ scoring

The response mean values and the proportions of participants with floor and ceiling effects by IADCQ item and IADCQ total are presented in Table 3. At the item level, the floor effects (0 = “Not at all”) ranged from 5% (item 12 on *Stress*) to 34.5% (item 8 on *Relationship with AD patient*), whereas the ceiling effects (4 = “Extremely”) ranged from 2.5% (item 1 on *Physical Health*) to 18% (item 12 on *Stress*). For the IADCQ total score, two participants had a floor effect (with sum score = 0) and another two participants had a ceiling effect (with sum score = 48). A total of 98% of the participants did not have either floor or ceiling effects, indicating that the IADCQ covers an ideal range of burden.

**Table 3:** **IADCQ item mean and percentage with floor and ceiling effects**

**IADCQ Question item**	**Item mean**	**With floor score (0 = "Not at All")**		**With ceiling score (4 = "Extremely")**	
**Concept**		**n**	%*	**n**	%*
Physical health	1.4	44	22.0%	5	2.5%
Impact on time	2.0	23	11.5%	17	8.5%
Sadness	2.0	16	8.0%	19	9.5%
Loneliness	1.5	50	25.0%	14	7.0%
Worry	2.0	34	17.0%	31	15.5%
Frustration	2.2	21	10.5%	35	17.5%
Social activities	2.1	23	11.5%	30	15.0%
Relationship with AD patient	1.3	69	34.5%	14	7.0%
Relationship with friends or family	1.4	49	24.5%	13	6.5%
Personal finances	1.5	54	27.0%	15	7.5%
Sleep	1.9	30	15.0%	22	11.0%
Stress	2.2	10	5.0%	36	18.0%
Entire scale (sum score 0–48)	21.6	2	1.0%	2	1.0%

Abbreviations: AD, Alzheimer’s disease; IADCQ, Impact of Alzheimer’s Disease on Caregiver Questionnaire.

*Percent calculated as n/N (200 Internet-survey participants).

### Classical psychometric evaluations

Because of the unidimensional model structure identified through CFA, the following item-level analyses were conducted (other item-level psychometrics are only appropriate in a multidimensional instrument): item-total correlations, alpha-removed statistics, and item homogeneity. The item-total correlations ranged from 0.523 (item 5 on *Worry*) to 0.785 (item 12 on *Stress*), which were considered as substantial and satisfactory to the hypothesized scale (Table 4) [35,38,39]. When alpha-if-item removed statistics were reviewed, removal of any one item did not lead to an appreciable improvement in coefficient alpha. Indeed, only item 5 (on *Worry*) had an improvement of any positive magnitude (0.001), but this improvement was negligible and far below the criterion of 0.02 improvement needed to flag the item as poor [31]. We have also noticed that the correlation of item 5 (*Worry*) was significantly different from the average correlation of the scale (*z* score = 2.7; *p* = 0.006). Nevertheless, CFA results were not as strong without item 5, providing a psychometric rationale for keeping it in the scale. No other items had item-total correlations that were significantly lower than the rest of the instrument’s average item-total correlation. In addition, the F_max_ value of 2.23 for the IADCQ indicates similar variances between the items.

**Table 4:** **Item-level psychometrics**

**IADCQ item**	**Concept**	**Correlation with total**	**Cronbach’s coefficient alpha**
Item 1	Physical health	0.672	0.921
Item 2	Impact on time	0.780	0.917
Item 3	Sadness	0.637	0.922
Item 4	Loneliness	0.701	0.920
Item 5*	*Worry*	*0.523*	*0.928*
Item 6	Frustration	0.729	0.919
Item 7	Social activities	0.723	0.919
Item 8	Relationship with AD patient	0.637	0.923
Item 9	Relationship with friends or family	0.763	0.918
Item 10	Personal finances	0.579	0.925
Item 11	Sleep	0.763	0.917
Item 12	Stress	0.785	0.917
Overall coefficient alpha			0.927

Abbreviation: AD, Alzheimer’s disease.

*The correlation of Item 5 is significantly different from the average correlation of the entire scale, where *z* score is 2.7 and *p* value is 0.006.

A series of scale-level psychometric evaluations were conducted (Table 5). Internal consistency reliability of the IADCQ revealed appropriate results: coefficient alpha was 0.927 and average inter-item correlation was 0.52. Reliability coefficients for the SF-12v2 scores were similar to published psychometrics for the general population [16]. The IADCQ had a mean scale score of 21.6 and a standard deviation of 10.8, which indicated that the majority of individuals within the Web-based participant population were likely to score along the scale continuum of 10.8 through 32.4. Convergent validity of the IADCQ scale was assessed by Pearson correlation coefficients with the SF-12v2 PCS and MCS scales and subscales. A low to moderate negative correlation was observed between the IADCQ and the scales of SF-12v2 with the Pearson correlation coefficients ranging from −0.58 to −0.20, which indicated a moderate convergent validity. Negative convergent correlations were expected here as higher scores on the IADCQ indicate worse functioning, whereas higher scores on the SF-12 indicate better functioning.

**Table 5:** **Scale-level psychometrics**

**Scale**	**Reliability**				
	**α**	**r**_***ii***_	Mean*	SD*	**Pearson** ***r*** with IADCQ*
IADCQ	0.927	0.52	21.62	10.77	**
SF-12 v2: PF	0.801	0.67	47.32	11.33	−0.32
SF-12 v2: RP	0.884	0.79	44.05	10.22	−0.42
SF-12 v2: BP (1 item)	**		45.32	11.26	−0.40
SF-12 v2: GH (1 item)	**		46.38	11.31	−0.20
SF-12 v2: VT (1 item)	**		45.84	10.41	−0.33
SF-12 v2: SF (1 item)	**		42.23	11.11	−0.54
SF-12 v2: RE	0.860	0.76	39.14	11.47	−0.48
SF-12 v2: MH	0.690	0.43	41.96	10.40	−0.57
SF-12 v2: PCS	0.875	0.56	48.18	10.43	−0.26
SF-12 v2: MCS	0.817	0.43	40.17	10.14	−0.58

Abbreviations: α, coefficient alpha; BP, bodily pain; GH, general health; IADCQ, Impact of Alzheimer’s Disease on Caregiver Questionnaire; MCS, mental health composite score; MH, mental health; PCS, physical health composite score; PF, physical functioning; r_*ii*_ , average inter-item correlation; RE, role emotional; RP, role physical; SD, standard deviation; SF, social functioning; SF-12v2, Short Form-12 Health Survey Version 2; VT, vitality.

*T-scores for SF-12v2 scales were used in calculation.

**Analyses were not conducted.

### Intra-class correlation coefficient

Intra-class correlation coefficient (ICC) for the IADCQ scale was estimated to assess the test-retest reliability for the subgroup of 50 AD caregivers who participated in the Web-based survey at both baseline and 4 weeks later. The ICC for the IADCQ scale was 0.68 (95% CI: 0.50–0.80), which indicated a moderate agreement on test-retest reliability.

## Discussion

The objective of this study was to investigate the psychometric characteristics of the IADCQ designed for AD caregivers as well as to determine the most appropriate scoring algorithm for the Web-based IADCQ. Our investigation revealed that the 12-item instrument demonstrated appropriate unidimensional model fit on the CFA, a high degree of item-level reliability, good internal consistency, and moderate test-retest reliability and moderate convergent validity with the scales of SF-12v2. Not surprisingly, negative correlations were observed because higher scores on the IADCQ indicate worse state with −0.20 for General Health and −0.58 for Mental Health. The CFA model was found to have strong fit on most of the indices. Moreover, most of the factor loadings were in the range of 0.7 to 0.8, indicating that the majority of the variance for question items was explained by the factor. The study findings demonstrate that the IADCQ can be used to measure the burden of AD caregiving and that the concepts measured in the IADCQ represent a cohesive concept of caregiver burden.

In addition to demonstrating psychometrically appropriate measurement characteristics, the results suggest that the IADCQ should be scored as a single scale by summing up the scores from all 12 items. The sum score implies the overall burden of AD caregiving across all theorized areas (ie, emotional, physical, social, financial, sleep, and time), where a higher score of the IADCQ indicates more burden for an AD caregiver. The IADCQ measures the burden of the AD caregiver; however, we recognize that there may be positive aspects associated with caregiving that are not addressed by our research. Positive emotions are not included in this newly developed measurement. Previous research has found additional factors to be important when discussing caregiving, such as positive emotions from caregiving and resources that caregivers may aid in managing their challenges of caregiving [40]. In particular, Stephan et al. have evaluated this aspect in the Caregiver Reaction Assessment scale [41]. Previous development work with the IADCQ did not consider these factors as the emphasis was on the negative side of caregiver burden. Readers should consider this omission of factors when evaluating the comprehensiveness of the IADCQ for their needs.

Unlike other caregiver instruments, the IADCQ has been specifically designed to measure the burden associated with caregiving for AD patients. Issues that AD caregivers in particular tend to face, such as the potential for the AD patient to harm him/herself or others and the relationship between the caregiver and the AD patient, are included in this instrument. This may allow for increased understanding of how caring specifically for a person with Alzheimer’s disease impacts the caregiver that other caregiver instruments may not adequately capture. Additionally, it is appropriate for use in a clinical trial setting in that it is self-administered, brief (12 easy-to-complete items), and simple to score and interpret.

The caregiver population interviewed in this study appears to be demographically similar to the AD caregiver population in the US. The 2009 Behavioral Risk Factor Surveillance System survey of caregivers of patients with AD and other dementias found 70% were female, 56% were ≥ 55 years old, and 44% were employed part or full time [4]. The Alzheimer’s Association reported 75% of caregivers had been caregivers for ≥ 1 year; of these, 32% had been caregivers for ≥ 5 years [5]. However, it should be noted that because the AD caregivers completed the questionnaire online, this may not represent those caregivers who do not have access to the Internet or are not computer users.

This study is not without limitations. As this study is the first quantitative evaluation of the IADCQ, its significance should not be overstated. The single-factor should be further validated with independent samples, such as samples of clinical trial subjects. Indeed, as the originally postulated construct of the IADCQ did not obtain appropriate fit, further validation of the unidimensional model is important. It is possible that the structure of the originally hypothesized model did not fit given a combination of too few items per factor and a sample size of only moderate power.

Additionally, the current research is limited to caregivers of AD. Extrapolating these findings to caregivers of dementia patients broadly is not advised based strictly on the current research. Both regulatory [42] and psychometric [43] guidance note that without proper assessment of the similarity of content validity, presuming a larger cohort (eg, dementia) will appropriately extrapolate to a more restrictive cohort from which the research is based (eg, AD) would be inappropriate. Therefore, we caution against any use of the IADCQ for a caregiver population of a broader dementia sample without additional research to establish such efficacy.

## Conclusions

In summary, this research supports the use of the Web-based IADCQ to measure the burden impact on caregivers of AD patients and justifies a single total-score interpretation. We found good internal consistency and moderate reliability and validity. Validation of the paper-based administration mode of the IADCQ is another area for future development. Additional psychometric evaluation should be further implemented because validity and reliability of an instrument in one administration mode (eg, Web-based survey) cannot be assumed to hold in an alternate mode (eg, paper-based survey) [37]. When the survey data are collected through other administration modes, additional psychometric properties of the instrument need to be assessed by other applicable approaches, such as development of a responder definition.

## Abbreviations

AD: Alzheimer’s disease
ADL: Activity of daily living
CFA: Confirmatory factor analysis
CFI: Comparative fit index
CI: Confidence interval
GFI: Goodness of fit index
HRQoL: Health-related quality of life
IADCQ: Impact of Alzheimer’s Disease on Caregiver Questionnaire
IADL: Instrumental activity of daily living
ICC: Intra-class correlation coefficient
MCS: Mental health composite score
NNFI: Non-normed fit index
RMSEA: Root mean square error of approximation
PCS: Physical health composite score
SAS: Statistical analysis software
SF-12v2: Short Form-12 Health Survey Version 2
SRMR: Standardized root mean square residual
US: United States
